# Supplementary material for: Origin of the Mobile Di-Hydro-Pteroate Synthase Gene Determining Sulfonamide Resistance in Clinical Isolates
Source: Front Microbiol. 2019 Jan 10;9:3332. doi: 10.3389/fmicb.2018.03332 (PMC6335563; doi:10.3389/fmicb.2018.03332)
Supplement: Supplementary file 2 [file Table_2.DOCX]

Supplementary Material

Origin of the mobile di-hydro-pteroate synthase gene determining sulfonamide resistance in clinical isolates

Miquel Sánchez-Osuna^1^, Pilar Cortés^1^, Jordi Barbé^1*^, Ivan Erill^2*^

*** Correspondence:** Corresponding Authors: [jordi.barbe@uab.cat](mailto:jordi.barbe@uab.cat); [erill@umbc.edu](mailto:erill@umbc.edu)

Supplementary Table 2 – List of oligonucleotides used in this work.

| **Primer Name** | **Sequence (5’ – 3’)** |
| --- | --- |
| folP_PlNde | CAGTcatatgTTCCAACGCCCGCGC |
| folP_PlBam | AGggatccTCATTGGCCCTGGGAGCGT |
| folP_LepNde | CAGTcatatgGAAAGCAAACAAGATAGT |
| folP_LepBam | AGggatccTTATTTGTTCATCAAATTCCAA |
| folP_ChlNde | CAGTcatatgACGAGCTGGAATTTTGT |
| folP_ChlBam | AGggatccTTACACGAACATCCCCGC |
| folP_RsNde | CAGTcatatgACCTATGTCCGTCCG |
| folP_RsBam | AGggatccTCATGCCTGCCTCCGTTC |
| sul2_Nde | CAGTcatatgAATAAATCGCTCATCAT |
| sul2_Bam | AGggatccTCAGCGCCGCCAATACCG |
