# Supplementary material for: Origin of the Mobile Di-Hydro-Pteroate Synthase Gene Determining Sulfonamide Resistance in Clinical Isolates
Source: Front Microbiol. 2019 Jan 10;9:3332. doi: 10.3389/fmicb.2018.03332 (PMC6335563; doi:10.3389/fmicb.2018.03332)
Supplement: Supplementary file 6 [file Data_Sheet_1.PDF]

Supplementary Material

Origin of the mobile di-hydro-pteroate synthase gene determining sulfonamide resistance in clinical isolates

Miquel Sánchez-Osuna[1], Pilar Cortés[1], Jordi Barbé[1*], Ivan Erill[2*]

*** Correspondence:** Corresponding Authors: jordi.barbe@uab.cat; erill@umbc.edu

Supplementary Data 1 - Multiple sequence alignment including *sul* genes at most 90% similar to those reported in the literature and one representative chromosomal *folP* gene from bacterial phyla with complete genomes available in RefSeq.

```
                                       10        20        30        40
                                ....|....|....|....|....|....|....|....|
Deferribacter_desulfuricans     ----------------------------------------
Elusimicrobium_minutum          ----------------------------------------
Caldisericum_exile              ----------------------------------------
Thermodesulfovibrio_yellowston  ----------------------------------------
Fusobacterium_nucleatum         ----------------------------------------
Bacillus_subtilis               ----------------------------------------
Dehalococcoides_mccartyi        ----------------------------------------
Dictyoglomus_turgidum           ----------------------------------------
Thermotoga_maritima             ----------------------------------------
Thermanaerovibrio_acidaminovor  ----------------------------------------
Coprothermobacter_proteolyticu  ----------------------------------------
Acidobacterium_capsulatum       ----------------------------------------
Streptomyces_coelicolor         ----------------------------------------
Kiritimatiella_glycovorans      ----------------------------------------
Rhodopirellula_baltica          ----------------------------------------
Fimbriimonas_ginsengisoli       ----------------------------------------
Deinococcus_radiodurans         ----------------------------------------
Desulfurispirillum_indicum      ----------------------------------------
Escherichia_coli                ----------------------------------------
Aquifex_aeolicus                ----------------------------------MLLIKH
Nostoc_punctiforme              ----------------------------------------
Caldithrix_abyssi               -----------------------------MQLRPILLNSR
```

```
Thermodesulfobacterium_geofont   ----------------------------------------
Treponema_succinifaciens         ----------------------------------------
Verrucomicrobium_spinosum        ----------------------------------------
Chlamydia_trachomatis            MTSWNFVCLSLGSNLGNRHEHIRRAYASLKKAGIRNLKSS
Sul2_Ecoli                       ----------------------------------------
SUL2 (PMID: 3075438)             ----------------------------------------
Sul2_Pmultocida                  ----------------------------------------
Sul2_Apleuropneumoniae           ----------------------------------------
Sul1_Ecloacae                    ----------------------------------------
SUL1 (PMID: 3054482)             ----------------------------------------
Sul1_Ecoli                       ----------------------------------------
Sul1_Uncultured                  ----------------------------------------
Sul3_Ecoli                       ----------------------------------------
SUL3 (PMID: 12604565)            ----------------------------------------
Parvibaculum_lavamentivorans     ----------------------------------------
Leptospira_interrogans           ----------------------------------------

                                          50        60        70        80
                                 ....|....|....|....|....|....|....|....|
Deferribacter_desulfuricans      --MFKIFEVKPDKNRLIFELKKIGVDDYALSLHEKGEILC
Elusimicrobium_minutum           ----------------------------------------
Caldisericum_exile               ----MIVRKIDFSYFECELEKVKVDSLVHNLFKEKSELIL
Thermodesulfovibrio_yellowston   ----------------------------------------
Fusobacterium_nucleatum          ----------------------------------------
Bacillus_subtilis                ----------------------------------------
Dehalococcoides_mccartyi         ----------------------------------------
Dictyoglomus_turgidum            -----MIVELSHEFLRKEILKVGAHPASLEIFERKAQIIP
Thermotoga_maritima              ----------------------------------------
Thermanaerovibrio_acidaminovor   --------MWDPRELQHRVKEVGADLRSVPYFMRKRGVLC
Coprothermobacter_proteolyticu   ----MVRLISKDVDLRELLNQIGSDPASYGIFLDKDSHLR
Acidobacterium_capsulatum        ----------------------------------------
Streptomyces_coelicolor          ----------------------------------------
Kiritimatiella_glycovorans       ----------------------------------------
Rhodopirellula_baltica           ----------------------------------------
Fimbriimonas_ginsengisoli        ----------------------------------------
Deinococcus_radiodurans          ----------------------------------------
Desulfurispirillum_indicum       ----------------------------------------
Escherichia_coli                 ----------------------------------------
```

```
Aquifex_aeolicus                  FEDKDLFYKFLKEKIGVFFREAEQRAFEGIFHCVYFSDPS
Nostoc_punctiforme                ----------------------------------------
Caldithrix_abyssi                 QRIKEINEKFNLKLTAEFTGSLFFKINDAEPFELEQLKQL
Thermodesulfobacterium_geofont    ----------------------------------------
Treponema_succinifaciens          ----------------------------------------
Verrucomicrobium_spinosum         ----------------------------------------
Chlamydia_trachomatis             VILETKALLLEGAPKEWDLPYFNSVVIGETQLSPDELIEE
Sul2_Ecoli                        ----------------------------------------
SUL2 (PMID: 3075438)              ----------------------------------------
Sul2_Pmultocida                   ----------------------------------------
Sul2_Apleuropneumoniae            ----------------------------------------
Sul1_Ecloacae                     ----------------------------------------
SUL1 (PMID: 3054482)              ----------------------------------------
Sul1_Ecoli                        ----------------------------------------
Sul1_Uncultured                   ----------------------------------------
Sul3_Ecoli                        ----------------------------------------
SUL3 (PMID: 12604565)             ----------------------------------------
Parvibaculum_lavamentivorans      ----------------------------------------
Leptospira_interrogans            ----------------------------------------

                                          90        100       110       120
                                  ....|....|....|....|....|....|....|....|
Deferribacter_desulfuricans       LKILNLTPGQANILKQEALAVGADVAVAKGAVTCKIEKTD
Elusimicrobium_minutum            ----------------------------------------
Caldisericum_exile                FKIYDLDARGANILKQEFLSAGGDVALSRDVASFRVEKSD
Thermodesulfovibrio_yellowston    ----------------------------------------
Fusobacterium_nucleatum           ----------------------------------------
Bacillus_subtilis                 ----------------------------------------
Dehalococcoides_mccartyi          ----------------------------------------
Dictyoglomus_turgidum             LKIFNISSPTANVIKQEMLSLGGDVVVHKNVVDCKIEKSD
Thermotoga_maritima               ----------------------------------------
Thermanaerovibrio_acidaminovor    LRLGPVDFRAANALKQELLARGGDAVVHGGAIAGTVYRTR
Coprothermobacter_proteolyticu    VWIDEVYYPAASVIKQECLSCGADAAVHKHVITGKVEKSS
Acidobacterium_capsulatum         ----------------------------------------
Streptomyces_coelicolor           ----------------------------------------
Kiritimatiella_glycovorans        ----------------------------------------
Rhodopirellula_baltica            ----------------------------------------
Fimbriimonas_ginsengisoli         ----------------------------------------
```

```
Deinococcus_radiodurans              ----------------------------------------
Desulfurispirillum_indicum           ----------------------------------------
Escherichia_coli                     ----------------------------------------
Aquifex_aeolicus                     IPPNALFESARKSCVSIFHKDGRTVVCGSEAKLKDFCSEL
Nostoc_punctiforme                   ----------------------------------------
Caldithrix_abyssi                    QRNGAFYFFRHDAQRNETLIVPDEMRFSQLSERNGGRDLP
Thermodesulfobacterium_geofont       ----------------------------------------
Treponema_succinifaciens             ----------------------------------------
Verrucomicrobium_spinosum            ----------------------------------------
Chlamydia_trachomatis                IKMIESRFGQDASLKWGPRPIDIDVLFYGDEAFSYHSDKC
Sul2_Ecoli                           ----------------------------------------
SUL2 (PMID: 3075438)                 ----------------------------------------
Sul2_Pmultocida                      ----------------------------------------
Sul2_Apleuropneumoniae               ----------------------------------------
Sul1_Ecloacae                        ----------------------------------------
SUL1 (PMID: 3054482)                 ----------------------------------------
Sul1_Ecoli                           ----------------------------------------
Sul1_Uncultured                      ----------------------------------------
Sul3_Ecoli                           ----------------------------------------
SUL3 (PMID: 12604565)                ----------------------------------------
Parvibaculum_lavamentivorans         ----------------------------------------
Leptospira_interrogans               ----------------------------------------

                                              130       140       150       160
                                     ....|....|....|....|....|....|....|....|
Deferribacter_desulfuricans          ALLLITKKSIRNLIRKLKLQPFNLDKLAIELEQLLKQKET
Elusimicrobium_minutum               ----------------------------------------
Caldisericum_exile                   AILIGTKKVYRKVLEKLAFMPYFGLRDVRVSLEKYLESTP
Thermodesulfovibrio_yellowston       --------------MKLKFHNF------------------
Fusobacterium_nucleatum              --------------MKMKKISCGN----------------
Bacillus_subtilis                    --------MAQHTIDQTQVIHTKP----------------
Dehalococcoides_mccartyi             ---------MDLPAKKPVESLFGS----------------
Dictyoglomus_turgidum                VILLGTKKHYELFIKKLENNKYFEIPLVLQELKEYLLKQK
Thermotoga_maritima                  ----------------------------------------
Thermanaerovibrio_acidaminovor       VILMGTPGQLRSLQEKLAHMPYFGLEEVRSGLKAALDNLD
Coprothermobacter_proteolyticu       VLILANVRQLKCLVDKLRRMPYWGLNQIADEIKSVLEDRE
Acidobacterium_capsulatum            -----------MAFRKRGQFEWPLRTRT------------
Streptomyces_coelicolor              ------------MSKQTGRRRRHLAG--------------
```

```
Kiritimatiella_glycovorans        -------------MTPPAEWICRGRR--------------
Rhodopirellula_baltica            -------------MMQRAVWRTSRRA--------------
Fimbriimonas_ginsengisoli         ----------------------------------------
Deinococcus_radiodurans           --MNWTGRIHELTFGFPVPSAEKGPNGW------------
Desulfurispirillum_indicum        --------------------MNLCAP--------------
Escherichia_coli                  -----MLRGFFLSIHTRDNIMKLFAQGT------------
Aquifex_aeolicus                  VKYPEAKKLAQEILESFIRYRKKYFQLNY-----------
Nostoc_punctiforme                -------------MPSNLIIRGRCFDW-------------
Caldithrix_abyssi                 LRLQEMIRARFDNSAPVWTVNHKTLD--------------
Thermodesulfobacterium_geofont    -------------MFPPLALGSKILD--------------
Treponema_succinifaciens          --------------MKTLNLASRKIS--------------
Verrucomicrobium_spinosum         ----------------------------------------
Chlamydia_trachomatis             TIPHPKVLERPFLLSMIASLCPYRRFRLEGSSCNGKTFAE
Sul2_Ecoli                        ----------------------------------------
SUL2 (PMID: 3075438)              ----------------------------------------
Sul2_Pmultocida                   ----------------------------------------
Sul2_Apleuropneumoniae            --------------------MKHKNRELVLFFAITFYYSL
Sul1_Ecloacae                     -----------------------------MGLCRYGAHNC
SUL1 (PMID: 3054482)              ----------------------------------------
Sul1_Ecoli                        ----------------------------------------
Sul1_Uncultured                   ----------------------------------------
Sul3_Ecoli                        ----------------------------------------
SUL3 (PMID: 12604565)             ----------------------------------------
Parvibaculum_lavamentivorans      ----------------------------------------
Leptospira_interrogans            ----------------------------------------

                                          170       180       190       200
                                  ....|....|....|....|....|....|....|....|
Deferribacter_desulfuricans       S-----YFVAKDVRLPLDKPYIVGILNVTPDSFSDGGKYL
Elusimicrobium_minutum            ----------------MKKPLIMGILNVTPDSFFDGGKTA
Caldisericum_exile                LP---IFEIRGRQFDFNSEKLIMGILNVTPDSFSDGGKFL
Thermodesulfovibrio_yellowston    ------------EFNFLKKTYIMGIINVTPDSFFDGGKYF
Fusobacterium_nucleatum           -----------KEIILGERTLVMGILNVTPDSFSDGGKYN
Bacillus_subtilis                 -----------SALSYKEKTLVMGILNVTPDSFSDGGKYD
Dehalococcoides_mccartyi          -----------KQFGWGERTYIMGIVNVTADSFSGDGLGA
Dictyoglomus_turgidum             PE--KIVSPWGRVLNLSR-TLVMGIINVTPDSFYSGSRKM
Thermotoga_maritima               -M--VYTTPWNRKIEFGR-TMVMGIINVTPDSFFADSRKQ
Thermanaerovibrio_acidaminovor    ISNWEVPLPGGRVLSLGRITKVMGIINLTPDSFFPGSRHQ
```

```
Coprothermobacter_proteolyticu  ARTFVLTMPNG-TLELRR-TKVMTILNATPDSFFPGSR-V
Acidobacterium_capsulatum       -------------LALGARTLVMGVLNVTPDSFSDGGLFL
Streptomyces_coelicolor         -------------LTAWDRCAVMGVVNVTPDSFSDGGRFF
Kiritimatiella_glycovorans      -------------VRTDGPPLVMGVLNVTPDSFSDGGRYL
Rhodopirellula_baltica          -------------LEIGRRPLVMGILNVTPDSFSDGGRFV
Fimbriimonas_ginsengisoli       -----------------MKLLLMGILNVTPDSFSDGGRYQ
Deinococcus_radiodurans         -------------TVRWPGCRVMGILNATPDSFSDGGQHL
Desulfurispirillum_indicum      ------------------WPKIAGILNVTPDSFSDGGCHL
Escherichia_coli                -------------SLDLSHPHVMGILNVTPDSFSDGGTHN
Aquifex_aeolicus                ---------NQKILPLGLKTAIMGVLNVTPDSFSDGGEFL
Nostoc_punctiforme              ----------------GQRTYLMGILNVTPDSFSDGGEFN
Caldithrix_abyssi               --------------FN-RGPLIMGILNVTPDSFSDGGRFY
Thermodesulfobacterium_geofont  --------------WK-NSPYFVGILNVTPDSFSDGGKYL
Treponema_succinifaciens        --------------TD-FPAFVMGIVNCTPDSFFSGSR--
Verrucomicrobium_spinosum       ----------------------MGIVNINDDSFSGDGSLD
Chlamydia_trachomatis           LAAIYPLTEEDALGSFGSATQIMGIVNITDNSISDTGLFL
Sul2_Ecoli                      ---------------MNKSLIIFGIVNITSDSFSDGGRYL
SUL2 (PMID: 3075438)            ---------------MNKSLIIFGIVNITSDSFSDGGRYL
Sul2_Pmultocida                 ---------------MNKSLIIFGIVNITSDSFSDGGRYL
Sul2_Apleuropneumoniae          FFYFKCSTDPIIEAPMNKSLIIFGIVNITSDSFSDGGRYL
Sul1_Ecloacae                   CLFARPIPIVEVAAEADAMVTVFGILNLTEDSFFDESRRL
SUL1 (PMID: 3054482)            ------------------MVTVFGILNLTEDSFFDESRRL
Sul1_Ecoli                      ------------------MVTVFGILNLTEDSFFDESRRL
Sul1_Uncultured                 -------------------MLPFCSPDPHRGSRCGGRRHG
Sul3_Ecoli                      ------------------MHTWLRFGEIMRGSLS------
SUL3 (PMID: 12604565)           ------------------MSKIFGIVNITTDSFSDGGLYL
Parvibaculum_lavamentivorans    ---------------MFQRPRIFGIVNITPDSFSDGGKYF
Leptospira_interrogans          ------MESKQDSNSSSYSTKIFGVLNITEDSFSDGGKYL

                                            210       220       230       240
                                ....|....|....|....|....|....|....|....|
Deferribacter_desulfuricans     GEK----------SIDARLDYFLENEIKIVDIGGESTRPG
Elusimicrobium_minutum          DP-------------FVRASKLIEEGADIIDIGGESTRPG
Caldisericum_exile              NI----------DDALKHAEQMVKEGADIIDVGGESTRPY
Thermodesulfovibrio_yellowston  NF----------QKAVDHAFRLIDEGADILDIGGESTRPG
Fusobacterium_nucleatum         NL----------DSAIKQAEKLILDGADIIDVGGESTRPG
Bacillus_subtilis               SL----------DKALLHAKEMIDDGAHIIDIGGESTRPG
Dehalococcoides_mccartyi        NP----------EAALEQARRFALEGADILDIGGESTRPG
```

```
Dictyoglomus_turgidum              QI----------NEVLKTVEDMVINGVDIIDIGGQSTRPG
Thermotoga_maritima                SV----------LEAVETAKKMIEEGADIIDVGGMSTRPG
Thermanaerovibrio_acidaminovor     GP----------DGALETAERMLSQGAHVLDLGAESTRPG
Coprothermobacter_proteolyticu     DL----------HTGLERAVDAEENGAAFIDIGGASTRPG
Acidobacterium_capsulatum          DT----------ARAVDQGLRMLEAGAAMVDVGGESTRPG
Streptomyces_coelicolor            DT----------TAAIKHGLDLVAQGADLVDVGGESTRPG
Kiritimatiella_glycovorans         DP----------DAAVEHALRMAADGAEIIDCGAESTRPG
Rhodopirellula_baltica             APGQQLTSSQTLELAVDAALKMQANGADLIDIGGESTRPY
Fimbriimonas_ginsengisoli          NA----------SDAVRFALQMIEDGADLIDVGGESTRPR
Deinococcus_radiodurans            QL----------DAALATARRMRDTGVFILDIGGESTRPG
Desulfurispirillum_indicum         QT----------RNAVERGLQMVVEGAHIIDVGGESTRPG
Escherichia_coli                   SL----------IDAVKHANLMINAGATIIDVGGESTRPG
Aquifex_aeolicus                   EP----------KKAVERAVKMAQEGAEIIDIGGESTRPG
Nostoc_punctiforme                 TT----------SAALVQAQALVAAGADIIDVGGQSTRPG
Caldithrix_abyssi                  ER----------DRAVERALQMEAEGALLIDIGGESTRPG
Thermodesulfobacterium_geofont     NM----------ESALKRVKELLEEGAEIIDVGGESTRPF
Treponema_succinifaciens           -------------GGAERAFELIGEGADILDLGGESTRPG
Verrucomicrobium_spinosum          AG-----------VALAQARQFLKAGADIIDIGAESARTN
Chlamydia_trachomatis              EAR----------RAAAHAERLFAEGASIIDLGAQATNPR
Sul2_Ecoli                         A----------PDAAIAQARKLMAEGADVIDLVRHPAIP-
SUL2 (PMID: 3075438)               A----------PDAAIAQARKLMAEGADVIDLVRHPAIP-
Sul2_Pmultocida                    A----------PDAAIAQARKLMAEGADVIDLGPASSNPD
Sul2_Apleuropneumoniae             A----------PDAAIAQARKLMAEGADVIDLGPASSNPD
Sul1_Ecloacae                      D----------PAGAVTAAIEMLRVGSDVVDVGPAASHPD
SUL1 (PMID: 3054482)               D----------PAGAVTAAIEMLRVGSDVVDVGPAASHPD
Sul1_Ecoli                         D----------PAGAVTAAIEMLRVGSDVVDVGPAASHPD
Sul1_Uncultured                    DGVRHSES---PAGAVTAAIEMLRVGSDVVDVGPAASHPD
Sul3_Ecoli                         ------------------ALHLVEDGADVIDLGAASSNPD
SUL3 (PMID: 12604565)              D----------TDKAIEHALHLVEDGADVIDLGAASSNPD
Parvibaculum_lavamentivorans       A----------ADAAIEHARNLASNGADVIDLGPASSNPD
Leptospira_interrogans             T----------FKSSSEKANSLLNQGADVIDIGAQSSNIQ
```

```
                                             250       260       270       280
                                   ....|....|....|....|....|....|....|....|
Deferribacter_desulfuricans        AEP-VSAKNEIKRIAYAIKRSL-------------EKG--
Elusimicrobium_minutum             AEP-VSFEDEKKRVIPVLKQIK-------------AAYPK
```

Caldisericum_exile	SES-VPLDEELRRVIPVIVAIR-------------KEFQT
Thermodesulfovibrio_yellowston	AEP-VSIDEELRRVIPVIEAIS-------------KRIS-
Fusobacterium_nucleatum	HVQ-ITSEEEISRVVPIIEKIS-------------KNLN-
Bacillus_subtilis	AEC-VSEDEEMSRVIPVIERIT-------------KELG-
Dehalococcoides_mccartyi	SQP-VNTREELDRVIPAVRLIS-------------RNLD-
Dictyoglomus_turgidum	AEP-VSIDEEMSRVIPAIESIR-------------KSYPD
Thermotoga_maritima	SDP-VDEEEELNRVIPVIRAIR--------------SITD
Thermanaerovibrio_acidaminovor	SDP-VSPEEELTRMLPPLRAIR-------------REFPE
Coprothermobacter_proteolyticu	AAE-IGEDEELRRVIPLLRQVR--------------ERVK
Acidobacterium_capsulatum	SHRTVSAQEEQDRVLPVIEAMR-------------KARPD
Streptomyces_coelicolor	ATR-VDEDEELRRVVPVVRGL---------------ASEG
Kiritimatiella_glycovorans	ARE-VPADEQIRRALPVLRSLA--------------RATD
Rhodopirellula_baltica	SDP-VDADIEIERVVPVIERLA--------------DRLA
Fimbriimonas_ginsengisoli	AEP-VSLDDELRRVLPVVEELS---------------AGG
Deinococcus_radiodurans	AEP-VDAATELDRVLPLIRALR---------------GEH
Desulfurispirillum_indicum	AAR-VSAEEQMKRILPVIDALV--------------A-HE
Escherichia_coli	AAE-VSVEEELQRVIPVVEAIA--------------QRFE
Aquifex_aeolicus	SKR-ISAEEELNRVLPALKEVR-------------RELPD
Nostoc_punctiforme	AKQ-ITLAEELDRVLSVLQVLR-------------PEIS-
Caldithrix_abyssi	AEP-VPEEEELRRVIPVIEKLR-------------SKS-D
Thermodesulfobacterium_geofont	SDP-VPEEEELKRVIPVIKAIK-------------SEFPE
Treponema_succinifaciens	SDY-VSAEEEIKRIVPVIEKIR-------------RKS-D
Verrucomicrobium_spinosum	RAA-ITEDEEVARLLPFLQAWPALTAEFASPPWDSDQLWP
Chlamydia_trachomatis	VKDLGSVEQEWERLEPVLRLLAERWG---------AAQQC
Sul2_Ecoli	TPR-LFRPTQKSRVCAGAGRAQ-------------ADG--
SUL2 (PMID: 3075438)	TPR-LFRPTQKSRVCAGAGRAQ-------------ADG--
Sul2_Pmultocida	AAP-VSSDTEIARIAPVLDALK-------------ADG--
Sul2_Apleuropneumoniae	AAP-VSSDTEIARIAPVLDALK-------------ADG--
Sul1_Ecloacae	ARP-VSPADEIRRIAPLLDALS-------------DQM--
SUL1 (PMID: 3054482)	ARP-VSPADEIRRIAPLLDALS-------------DQM--
Sul1_Ecoli	ARP-VSPADEIRRIAPLLDALS-------------DQM--
Sul1_Uncultured	ARP-VSPADEIRRIAPLLDALS-------------DQM--
Sul3_Ecoli	TTE-VGVVEEIKRLKPVIKALK-------------EKG--
SUL3 (PMID: 12604565)	TTE-VGVVEEIKRLKPVIKALK-------------EKG--
**Parvibaculum_lavamentivorans**	AVP-VPPAEEIRRLGPVIEALA-------------GDG--
**Leptospira_interrogans**	ASL-IGPEIEWIRMKELISNLV-------------AKG--
	290 300 310 320

```
                                   ....|....|....|....|....|....|....|....|
Deferribacter_desulfuricans        FVVSVDTYKSVVAEFALKEG-VHIINDISGFNFDN--NMA
Elusimicrobium_minutum             ITISIDTYKPKIAKLAVEEG-ADIINDPSGL-ADP--VMA
Caldisericum_exile                 IPISIDTYKSKVAEEAINAG-ADIINDISGLRFDP--LMI
Thermodesulfovibrio_yellowston     IPISIDTYKARVAEEAIKAG-ATIINDISGLRFDP--QMS
Fusobacterium_nucleatum            TIISIDTYKYDVAEEAIKAG-ANIINDIWGLQYDNG-EMA
Bacillus_subtilis                  VPISVDTYKASVADEAVKAG-ASIINDIWGAKHDP--KMA
Dehalococcoides_mccartyi           IPVSVDTYRREVAEEALKAG-ACIINDVWGLKKDP--SLF
Dictyoglomus_turgidum              VVISVDTYYSKVAELAVDRG-ADMINDISAFRFDN--DLV
Thermotoga_maritima                VPISVDTYRWRVALKALEAG-ADIVNDISGYQFEP--DIV
Thermanaerovibrio_acidaminovor     AVISVDTYRSATARACAAEG-ADMVNDISGLSFDP--EMA
Coprothermobacter_proteolyticu     LFISVDTYRAEVAKQALENG-ADLINDIYGLQFDK--EMA
Acidobacterium_capsulatum          AVISIDTYKAATAAACLRAG-AEIVNDVSGFLWDE--AMP
Streptomyces_coelicolor            VTVSVDTMRASVAGQALAAG-ATLVNDVSGGLADP--RMI
Kiritimatiella_glycovorans         ALISIDTRSAVVAREALGAG-AHIVNDVSALRHDP--AMA
Rhodopirellula_baltica             IPISVDTSKATVANAAIQAG-AEIVNDVSGLEGDP--EMP
Fimbriimonas_ginsengisoli          IQVSVDTMKPEVARRALAAG-ARVINDVTALR-EP--EMR
Deinococcus_radiodurans            VLLSVDTMKPEVAAEALRAG-AHLVNDVTGLR-DP--AMI
Desulfurispirillum_indicum         VPISVDTTSALVARVCIDRG-ATMINDISAGKDDP--AMF
Escherichia_coli                   VWISVDTSKPEVIRESAKVG-AHIINDIRS-LSEP--GAL
Aquifex_aeolicus                   TWISVDTYKAEVAKACLDEG-ADIINDVSGGTFDP--EIL
Nostoc_punctiforme                 VPISVDTTRAAVAKASVEAG-ADIINDISGGTFDS--EML
Caldithrix_abyssi                  VLISVDTYKSSIARAALKAG-ADIVNDISAAQFDD--KMI
Thermodesulfobacterium_geofont     AIISIDTYKSKVAEEALKAG-ANIVNDISALRFDP--KMI
Treponema_succinifaciens           IPISVDTRKKSVMQAAFNSG-ADILNDISALEDEP--ELA
Verrucomicrobium_spinosum          PILSINTWRSGVIERVLPLG-GDLINDISGLPDAR---NA
Chlamydia_trachomatis              PDVSIDTFRPEIIRRAVEVFPIRWINDVSGGS----LEMA
Sul2_Ecoli                         IPVSLDSYQPATQAYALSRG-VAYLNDIRGFPDA---AFY
SUL2 (PMID: 3075438)               IPVSLDSYQPATQAYALSRG-VAYLNDIRGFPDA---AFY
Sul2_Pmultocida                    IPVSLDSYQPATQAYALSRG-VAYLNDIRGFPDT---AFY
Sul2_Apleuropneumoniae             IPVSLDSYQPATQAYALSRG-VAYLNDIRGFPDA---AFY
Sul1_Ecloacae                      HRVSIDSFQPETQRYALKRG-VGYLNDIQGFPDP---ALY
SUL1 (PMID: 3054482)               HRVSIDSFQPETQRYALKRG-VGYLNDIQGFPDP---ALY
Sul1_Ecoli                         HRVSIDSFQPETQRYALKRG-VGYLNDIQGFPDP---ALY
Sul1_Uncultured                    HRVSIDSFQPETQRYALKRG-VGYLNDIQGFPDP---ALY
Sul3_Ecoli                         ISISVDTFKPEVQSFCIEQK-VDFINDIQGFPYP---EIY
SUL3 (PMID: 12604565)              ISISVDTFKPEVQSFCIEQK-VDFINDIQGFPYP---EIY
Parvibaculum_lavamentivorans       IPLSVDSFHVETQAFALAHG-AAYLNDIQGFAEP---ADY
```

```
Leptospira_interrogans           VRISVDSFQPQVIANSLSAG-VEFINHIRGFVDEESIREI

                                          330       340       350       360
                                 ....|....|....|....|....|....|....|....|
Deferribacter_desulfuricans      EVCARYGCGVCLMHIKGTPKN--MQDN--PTYNNLLEDIK
Elusimicrobium_minutum           DIAASSDTKLVVMHTRGTPQN--MDKL--TEYADIVADIK
Caldisericum_exile               DVAKRYNVPVVIMHIKGTPKD--MQKN--PVYEDLMRELL
Thermodesulfovibrio_yellowston   TIASKYKVPVIVMHIKGTPKD--MQKN--PYYEALIPEII
Fusobacterium_nucleatum          ELVKKSKLPIIAMHNQ--------NDE--IYSKDIMLSLR
Bacillus_subtilis                SVAAEHNVPIVLMHNR--------PER--NYN-DLLPDML
Dehalococcoides_mccartyi         ELAAKRPAGWVITSNQ--------RE---NPAEDIMAAVT
Dictyoglomus_turgidum            KIVAKYKVPYILMHMKGTPKD--MQKN--PYYDDVVREIM
Thermotoga_maritima              RVVSENNVPYVLMHIKGTPKT--MQEN--PHYEDVVKEIK
Thermanaerovibrio_acidaminovor   RTVADSGCALVLMHIKGRPKD--MQQN--PTYRDLLGEIS
Coprothermobacter_proteolyticu   NVIADYGVPVVIMHMRGTPAT--MQEY--ASYDDLMKELH
Acidobacterium_capsulatum        ALCAAGACGVVLMHTRGTPNEWRGLPK--LEGDEVLPLVR
Streptomyces_coelicolor          PVVADTGAPFVVMHWRGLLKD--GNVK--GTYADVVTEVV
Kiritimatiella_glycovorans       ATAAEFEAGVVLMHMRGTPET--MQRE--TGYTDVAGEVC
Rhodopirellula_baltica           AVVVESLAGVCVMHMQGNPQT--MQDD--PSYGDVVEEIE
Fimbriimonas_ginsengisoli        EICAGAECTVCLMHMKGEPRT--MQES--PAYEDVVSEVR
Deinococcus_radiodurans          RVCADAGAAACVMHMQGEPRT--MQHQ--PVYSDVVAEVH
Desulfurispirillum_indicum       PLAVEREVDICLMHKRGNPAV--MQQL--THYDDVVDEVH
Escherichia_coli                 EAAAETGLPVCLMHMQGNPKT--MQEA--PKYDDVFAEVN
Aquifex_aeolicus                 KVVTEYRCPYVINHMKGRPET--WKEE-PIIYEDVVEEIS
Nostoc_punctiforme               PTVAELSVPIILMHIRGTPQT--MQQQ--TDYQDLLGEIS
Caldithrix_abyssi                EVVRDFDCPIIIMHIKGTPQN--MQNN--PYYEDVVEEVY
Thermodesulfobacterium_geofont   EVVRDFGCPIIIMHMQGNPKT--MQIN--PTYKDVVKEIK
Treponema_succinifaciens         FFAAEKKIPVILMHKRGIPST--MQAD--TEYKNIFNEVS
Verrucomicrobium_spinosum        ELCAQHDAALLIMHSVGEPK----IPHTHVSYPDVMETLD
Chlamydia_trachomatis            HLAKEFGLRLLINHSCSLPPR---PDCVLSYEESPIEQML
Sul2_Ecoli                       PQLAKSSAKLVVMHSVQD-GQ--ADRR-EAPAGDIMDHIA
SUL2 (PMID: 3075438)             PQLAKSSAKLVVMHSVQD-GQ--ADRR-EAPAGDIMDHIA
Sul2_Pmultocida                  PQLAKSSAKLVVMHSVQD-GQ--ADRR-EAPAGDIMDHIA
Sul2_Apleuropneumoniae           PQLAKSSAKLVVMHSVQD-GQ--ADRR-EAPAGDIMDHIA
Sul1_Ecloacae                    PDIAEADCRLVVMHSAQRDGI--ATRTGHLRPEDALDEIV
SUL1 (PMID: 3054482)             PDIAEADCRLVVMHSAQRDGI--ATRTGHLRPEDALDEIV
Sul1_Ecoli                       PDIAEADCRLVVMHSAQRDGI--ATRTGHLRPEDALDEIV
Sul1_Uncultured                  PDIAEADCRLVVMHSAQRDGI--ATRTGHLRPEDALDEIV
```

```
Sul3_Ecoli                          SGLAKSDCKLVLMHSVQRIGA--ATKV-ETNPEEVFTSMM
SUL3 (PMID: 12604565)               SGLAKSDCKLVLMHSVQRIGA--ATKV-ETNPEEVFTSMM
Parvibaculum_lavamentivorans        PALAATDARLVLMHSIQGRGN--ADRR-SAPEGDILDHIG
Leptospira_interrogans              SKYARTNRKFILMYSHNHSNR--AETKSHLTPNNVILEIV

                                            370       380       390       400
                                    ....|....|....|....|....|....|....|....|
Deferribacter_desulfuricans         LYLKKSIDIALKCGVSK-NSIMLDPGFGF--GKTLDDNYL
Elusimicrobium_minutum              NFFEKKIQECAREGLHT-DNIILDPGFGF--AKNKEQNFL
Caldisericum_exile                  QYFEERITFLNSVGIDK---IVIDPGIGF--GKTREHNLE
Thermodesulfovibrio_yellowston      EYLRDSVVIAKQAGVDE-NMIIIDPGIGF--GKLPEHNLT
Fusobacterium_nucleatum             EFFEKTYKIADKYGIDR-DKIILDPGLGF--GKNVEQNIE
Bacillus_subtilis                   SDLMESVKIAVEAGVDE-KNIILDPGIGF--AKTYHDNLA
Dehalococcoides_mccartyi            SELRETALRLLGLGVPA-RNIIIDPGVGF--GKTLEQNLE
Dictyoglomus_turgidum               EFFEERINYAKENGVDP-EKIIVDPGIGF--GKRYEDNLE
Thermotoga_maritima                 EYFTEKIEYLKEKGVN---QIVLDPGIGF--GKRYEDNLE
Thermanaerovibrio_acidaminovor      DFFEHQMDLAERGGIPR-DRIILDPGIGF--GKTYQHNLE
Coprothermobacter_proteolyticu      TYFVERVEYALSKGIKE-NQIILDPGIGF--AKLPEHNLE
Acidobacterium_capsulatum           DGLRERLAAAMQAGVAA-ERVALDPGYGF--GKVLGENYS
Streptomyces_coelicolor             DELHARVDAVLDGGIAP-DRVIVDPGLGF--SKDAEHDLV
Kiritimatiella_glycovorans          AHLERRVHAAREAGIAE-TRIAVDPGIGF--GKTAEQNVT
Rhodopirellula_baltica              RYLLARRQACLDLGIEP-ERICLDPGIGF--GKTHDHNLT
Fimbriimonas_ginsengisoli           EFLLGAAHQAEAAGVKR-EKIWLDPGIGF--GKTVEHNLA
Deinococcus_radiodurans             GFLRERAAEVKSAGVP---SVLLDPGIGF--GKTLDHNLS
Desulfurispirillum_indicum          HYLAMRAQAFLEAGGSA-GRLFLDPGIGL--PKDSQANLR
Escherichia_coli                    RYFIEQIARCEQAGIAK-EKLLLDPGFGF--GKNLSHNYS
Aquifex_aeolicus                    QFFKNQINKLKELGFREEEKIILDPGIGF--GKLPEHNVE
Nostoc_punctiforme                  SFLARQVQAATTAGI-DLDKIIIDPGIGF--AKNYEQNLE
Caldithrix_abyssi                   HYFEERIERLERAGIG---KIIIDPGIGF--GKRLEDNLH
Thermodesulfobacterium_geofont      EFLKKRIEFLVKKGIPF-ENIIIDPGIGF--GKTFSHNLQ
Treponema_succinifaciens            SYLEQRAEFAIKNGIEK-EKIIVDPGIGF--GKNLEGNLN
Verrucomicrobium_spinosum           RFFEDKLLLAKTAGLSE-ERVLLDPGIDF--AKQRDDNLA
Chlamydia_trachomatis               RWGESQLEQFAQVGLDTSWQVVFDPGIGF--GKTPVQSML
Sul2_Ecoli                          AFFDARIAALTGAGIKR-NRLVLDPGMGFFLGAAPETSLS
SUL2 (PMID: 3075438)                AFFDARIAALTGAGIKR-NRLVLDPGMGFFLGAAPETSLS
Sul2_Pmultocida                     AFFDARIAALTGAGIKR-NRLVLDPGMGFFLGAAPETSLS
Sul2_Apleuropneumoniae              AFFDARIAALTGAGIKR-NRLVLDPGMGFFLGAAPETSLS
Sul1_Ecloacae                       RFFEARVSALRRSGVAA-DRLILDPGLGFFLSPAPETSLH
```

```
SUL1 (PMID: 3054482)              RFFEARVSALRRSGVAA-DRLILDPGMGFFLSPAPETSLH
Sul1_Ecoli                        RFFEARVSALRRSGVAA-DRLILDPGMGFFLSPAPETSLH
Sul1_Uncultured                   RFFEARVSALRRSGVAA-DRLILDPGMGFFLSPAPETSLH
Sul3_Ecoli                        EFFKERIAALVEAGVKR-ERIILDPGMGFFLGSNPETSIL
SUL3 (PMID: 12604565)             EFFKERIAALVEAGVKR-ERIILDPGMGFFLGSNPETSIL
Parvibaculum_lavamentivorans      RFFEARLAALEQAGIAR-ARVILDPGMGFFVGPRPETSFS
Leptospira_interrogans            RFFRERKKILLNAGIAQ-EQLIFDPGMGFFLSSDFQVSFE

                                          410       420       430       440
                                  ....|....|....|....|....|....|....|....|
Deferribacter_desulfuricans       ILKYLEEIKTLGY-PLFIGVSRKSMIGKIIDK--------
Elusimicrobium_minutum            LLKNISYYKSLGL-PLLIGLSRKKFLAKEGD---------
Caldisericum_exile                ILNKLQEFTIFGK-PVLVGLSRKSFIGLTLD----NR---
Thermodesulfovibrio_yellowston    IIKNLREFSNLGK-PLLIGVSRKSFIGKVLN----ES---
Fusobacterium_nucleatum           VLSRLNELKDMG--SILLGASKKRFIGKLLN----DL---
Bacillus_subtilis                 VMNKLEIFSGLGY-PVLLATSRKRFIGRVL-----DL---
Dehalococcoides_mccartyi          IVRRLSELKTLNL-PILLGTSRKSLIGQVL-----DT---
Dictyoglomus_turgidum             IMARLKEFKSLRK-PILIGASRKSFIGKALS----DL---
Thermotoga_maritima               ILRRIDEFKELKL-PILIGASRKSFIGITLG----NV---
Thermanaerovibrio_acidaminovor    ILRQLEAFSTHGR-PILIGASRKSTVGIATD----SK---
Coprothermobacter_proteolyticu    VLRRIEELFTLGF-PVLVGHSRKSTMGKILG----GV---
Acidobacterium_capsulatum         LLAGQDALLELGR-PLLVGVSRKSFLRKTLER---ELAHL
Streptomyces_coelicolor           LLAHLDRLLTLGH-PLLVAASRKRFLGRVLAG---PDAAP
Kiritimatiella_glycovorans        LLRALPRLVESGR-PVLVGASRKSFIGRLLDR---PAG--
Rhodopirellula_baltica            LLRATSRFASLGS-PILIGHSRKGFIRKVLDR---NERCT
Fimbriimonas_ginsengisoli         LLRNLDKLVETGY-PVLVGVSRKSFLGKFSGT---GVL--
Deinococcus_radiodurans           LLRAVDDLASGPD-PVLIAASRKKTIDLIAG-----VP--
Desulfurispirillum_indicum        LIMSMGRLRQLGY-QTYLGASRKSFIGAICQV---EDP--
Escherichia_coli                  LLARLAEFHHFNL-PLLVGMSRKSMIGQLLNV---G-P--
Aquifex_aeolicus                  ILKRFHEFKIFGK-ILMVGVSRKSFIGLILEGFLNRKT--
Nostoc_punctiforme                IFRGLRSLTTLNC-PILVGASRKSFIGRILN-----QP--
Caldithrix_abyssi                 LLRDLKDFTFLQR-PILMGTSRKSFIGKILNK--------
Thermodesulfobacterium_geofont    ILKNLDSFKELNR-PLMIGHSRKSFIGEIINK--------
Treponema_succinifaciens          LISNCGSLCGGKF-PVLMALSRKSCIGQVTGR--------
Verrucomicrobium_spinosum         IYRELDRLHRFGR-PLLLPVSRKTVIGQVLELP-------
Chlamydia_trachomatis             LMDGVKQFKRVLECPVLIGHSRKSCLS-MLGR--------
Sul2_Ecoli                        VLARFDELRLRFDLPVLLSVSRKSFLR-ALTG--------
SUL2 (PMID: 3075438)              VLARFDELRLRFDLPVLLSVSRKSFLR-ALTG--------
```

```
Sul2_Pmultocida                      VLARFDELRLRFDLPVLLSVSRKSFLR-ALTG--------
Sul2_Apleuropneumoniae               VLARFDELRLRFDLPVLLSVSRKSFLR-ALTG--------
Sul1_Ecloacae                        VLSNLQKLKSALGLPLLVSVSRKSFLG-ATVG--------
SUL1 (PMID: 3054482)                 VLSNLQKLKSALGLPLLVSVSRKSFLG-ATVG--------
Sul1_Ecoli                           VLSNLQKLKSALGLPLLVSVSRKSFLG-ATVG--------
Sul1_Uncultured                      VLSNLQKLKSALGLPLLVSVSRKSFLG-ATVG--------
Sul3_Ecoli                           VLKRFPEIQEAFNLQVMIAVSRKSFLG-KITG--------
SUL3 (PMID: 12604565)                VLKRFPEIQEAFNLQVMIAVSRKSFLG-KITG--------
Parvibaculum_lavamentivorans         ILARLGELKERFGLPVFISVSRKSFLR-AVTG--------
Leptospira_interrogans               VLKKIKTLQEEFS-PMMVSVTKKSFLGNALGG--------

                                             450       460       470       480
                                     ....|....|....|....|....|....|....|....|
Deferribacter_desulfuricans          ---TPEERDVPSKAIEFLAMIKGANFIRTHEVKYAKDIAK
Elusimicrobium_minutum               ---TPADRLEATLSANLYAAMNGADILRVH------DVAE
Caldisericum_exile                   ---PVEERLYGTLASNMFALLKGASILRVHDVLPHKDMIR
Thermodesulfovibrio_yellowston       ---LPEKRLEGTASAVAISVINGANIVRVHDVGFMAKVVK
Fusobacterium_nucleatum              ---PFDERVEGTVATTVIGIEKGVDIVRVHNVLENKRACL
Bacillus_subtilis                    ---PPEERAEGTGATVCLGIQKGCDIVRVHDVKQIARMAK
Dehalococcoides_mccartyi             ---PPGERLEGTAATVALGIGGGADIVRVHDVAYMKRVCR
Dictyoglomus_turgidum                ---PPEERLEGTLGITALCVLNDVDIVRVHDVKENKRVIK
Thermotoga_maritima                  ---PPEERLEGTLAVTAYCTMKGVDIIRVHDVLPNKRVIR
Thermanaerovibrio_acidaminovor       ---DPEDRLEGTLAITSLCAMRGIALVRVHDVEENVKTIK
Coprothermobacter_proteolyticu       ---PAEERLFGTVAWTSYLTWKGVHIVRVHDTKPNVDAVK
Acidobacterium_capsulatum            LREPDDDLTTATMAACTAAVLGGAHIVRVHDVAPALAAVA
Streptomyces_coelicolor              P--PARERDAATAAVSALAAQAGAWAVRVHEVRATADAVR
Kiritimatiella_glycovorans           ------ERMAGGLAVALHAAARGARIIRTHDVRETCDALR
Rhodopirellula_baltica               K--DDYNPMAGTLAVSMAVAAAGAHVIRVHDVAETVQALD
Fimbriimonas_ginsengisoli            ---PVEERLPGTLAAQVLAQAQGARIIRAHDVLEARRAID
Deinococcus_radiodurans              ---EAADRDPGSLALHLHAARCGAALVRLHAAGEMVQALR
Desulfurispirillum_indicum           -----RERLPGSLVVAMEAYRQGVHMLRVHDVAATVQALK
Escherichia_coli                     -----SERLSGSLACAVIAAMQGAHIIRVHDVKETVEAMR
Aquifex_aeolicus                     ---EPKERLFGSLGALAPAVLGGASIVRVHDVKETREFLA
Nostoc_punctiforme                   ---DPKARVWGTAAACCTAIFNGADILRVHDVQEMHDVSL
Caldithrix_abyssi                    ---EVDERIYGSLATQILAVQNGANIVRVHDVQATQDALK
Thermodesulfobacterium_geofont       ---PPSLRDGGTVGVSLFAYLKGVQFLRVHKVDINKDALS
Treponema_succinifaciens             ---KVQDRLFGTLAADLISVLKGAFMVRVHDIAPCKDTLA
Verrucomicrobium_spinosum            ---NPQDRDAGTVACIAVGIRRGAQIFRVHHVEAAAQAVK
```

```
Chlamydia_trachomatis                --FNSNDRDWETIGCSVSLHDRGVDYLRVHQVEGNRRALA
Sul2_Ecoli                           --RGPGVSGPRHSLQSLPPPQVELTSSAHTSRAPCATGWR
SUL2 (PMID: 3075438)                 --RGPGVSGPRHSLQSLPPPQVELTSSAHTSRAPCATGWR
Sul2_Pmultocida                      --RGPGDVGAATLAAELAAAAGGADFIRTHEPRPLRDGLA
Sul2_Apleuropneumoniae               --RGPGDVGAATLAAELAAAAGGADFIRTHEPRPLRDGLA
Sul1_Ecloacae                        --LPVKDLGPASLAAELHAIGNGADYVRTHAPGDLRSAIT
SUL1 (PMID: 3054482)                 --LPVKDLGPASLAAELHAIGNGADYVRTHAPGDLRSAIT
Sul1_Ecoli                           --LPVKDLGPASLAAELHAIGN------------------
Sul1_Uncultured                      --LPVKDLGPASLAAELHAIGNGADYVRTHAPGDLRSAIT
Sul3_Ecoli                           --TDVKSRLAPTLAAEMYAYKKGADYLRTHDVKSLSDALK
SUL3 (PMID: 12604565)                --TDVKSRLAPTLAAEMYAYKKGADYLRTHDVKSLSDALK
Parvibaculum_lavamentivorans         --RAPGEAGAATLAAELMAALNGADFIRTHEPAPLLDAFS
Leptospira_interrogans               --LKVEEREIPTVIAELYLCIQNVEYIRTHEPKNLKQALK

                                             490       500
                                     ....|....|....|....|....
Deferribacter_desulfuricans          IAEY---YSKVELNA---------
Elusimicrobium_minutum               TIKI---LEKIPVS----------
Caldisericum_exile                   MYKA---IVSEGR-----------
Thermodesulfovibrio_yellowston       MADA---IKFS-------------
Fusobacterium_nucleatum              VADG---IYRKRG-----------
Bacillus_subtilis                    MMDA---MLNKGGVHHG-------
Dehalococcoides_mccartyi             MSDA---VVRGKIWK---------
Dictyoglomus_turgidum                VLEE---IKCIRSSSL--------
Thermotoga_maritima                  MMEA---ILWQRL-----------
Thermanaerovibrio_acidaminovor       MIEA---VKEAGL-----------
Coprothermobacter_proteolyticu       AVEA---IREGI------------
Acidobacterium_capsulatum            IADE---VLRAG------------
Streptomyces_coelicolor              VARA---VEGAR------------
Kiritimatiella_glycovorans           IWAT---LAREGKE----------
Rhodopirellula_baltica               LFEASGGLEVSFNDRTI-------
Fimbriimonas_ginsengisoli            VASA---ILGYPAGSISAV-----
Deinococcus_radiodurans              VEAA---LA---------------
Desulfurispirillum_indicum           MAAAL--WAHTDNNKL--------
Escherichia_coli                     VVEAT--LSAKENKRYE-------
Aquifex_aeolicus                     LLDA---VRTYDVS----------
Nostoc_punctiforme                   VADA---LLRQPIQHDC-------
Caldithrix_abyssi                    ILKA---VQYFETD----------
```

Thermodesulfobacterium_geofont	TFKF---LIEN-------------
Treponema_succinifaciens	VLKS---LLKYESV----------
Verrucomicrobium_spinosum	VLWQATHPSPIATSAA--------
Chlamydia_trachomatis	AAAWAGMFV---------------
Sul2_Ecoli	YWRR--------------------
SUL2 (PMID: 3075438)	YWRR--------------------
Sul2_Pmultocida	VLAALKETARIR------------
Sul2_Apleuropneumoniae	VLAALKETARIRYQKTEGTSIESN
Sul1_Ecloacae	FSETLAKFRSRDARDRGLDHA---
SUL1 (PMID: 3054482)	FSETLAKFRSRDARDRGLDHA---
Sul1_Ecoli	------------------------
Sul1_Uncultured	FSETLAKFRSRDARDRGLDHA---
Sul3_Ecoli	ISKALG------------------
SUL3 (PMID: 12604565)	ISKALG------------------
**Parvibaculum_lavamentivorans**	VAACVEAGRSQGQ-----------
**Leptospira_interrogans**	IWNLMNK-----------------
