# Supplementary material for: Origin of the Mobile Di-Hydro-Pteroate Synthase Gene Determining Sulfonamide Resistance in Clinical Isolates
Source: Front Microbiol. 2019 Jan 10;9:3332. doi: 10.3389/fmicb.2018.03332 (PMC6335563; doi:10.3389/fmicb.2018.03332)
Supplement: Supplementary file 7 [file Data_Sheet_2.PDF]

Supplementary Material

Origin of the mobile di-hydro-pteroate synthase gene determining sulfonamide resistance in clinical isolates

Miquel Sánchez-Osuna[1], Pilar Cortés[1], Jordi Barbé[1*], Ivan Erill[2*]

*** Correspondence:** Corresponding Authors: jordi.barbe@uab.cat; erill@umbc.edu

Supplementary Data 2 – PROSITE-formatted pattern of the region containing the identified two-amino acid insertion in *sul*-encoded proteins used to seed the PHI-BLAST search.

A-L-x(2)-[AS]-G-[IV]-x(2)-[DEN]-R-[IL]-[IV]-L-D-P-G-[LM]-G-F-F-L-[GS]-[APS]-[AN]-P-E-T-S-[IL]-x-V-L-x-[NR]-[FL]-[DPQ]-[EK]-[IL]-x(3)-[FL]
