# Supplementary material for: Origin of the Mobile Di-Hydro-Pteroate Synthase Gene Determining Sulfonamide Resistance in Clinical Isolates
Source: Front Microbiol. 2019 Jan 10;9:3332. doi: 10.3389/fmicb.2018.03332 (PMC6335563; doi:10.3389/fmicb.2018.03332)
Supplement: Supplementary file 8 [file Data_Sheet_3.PDF]

Supplementary Material

Origin of the mobile di-hydro-pteroate synthase gene determining sulfonamide resistance in clinical isolates

Miquel Sánchez-Osuna[1], Pilar Cortés[1], Jordi Barbé[1*], Ivan Erill[2*]

*** Correspondence:** Corresponding Authors: jordi.barbe@uab.cat; erill@umbc.edu

```
Leptospira santarosai            VLLNEGITQE-QLIFDPGMGFFLSPDFQVSFEVLRKI
Leptospira interrogans           ILLNAGIAQE-QLIFDPGMGFFLSSDFQVSFEVLKKI
Leptospira alstonii              EMLESGISQE-QLIFDPGMGFFLSPDFQISFEVLRKI
Leptospira biflexa               DLTAMGISES-SIFFDPGMGFFLSDDPMVSFRVLQDL
Sul2 Ecoli 2                     ALTGAGIKRN-RLVLDPGMGFFLGAAPETSLSVLARF
Sul2 Apleuropneumoniae           ALTGAGIKRN-RLVLDPGMGFFLGAAPETSLSVLARF
Sul2 Ecoli 1                     ALTGAGIKRN-RLVLDPGMGFFLGAAPETSLSVLARF
Parvibaculum lavamentivorans     ALEQAGIARA-RVILDPGMGFFVGPRPETSFSILARL
Candidatus Phaeomarinobacter     GLAASGIARE-RLVLDPGMGFFLGTDPEVSLEVLRHV
Sul1 Cglutamicum                 ALRRSGVAAD-RLILDPGMGFFLSPAPETSLHVLSNL
Sul1 Ecloacae                    ALRRSGVAAD-RLILDPGLGFFLSPAPETSLHVLSNL
Sul3 Ecoli                       ALVEAGVKRE-RIILDPGMGFFLGSNPETSILVLKRF
Sul3 SCholeraesuis               ALVEAGVKRE-RIILDPGMGFFLGSNPETSILVLKRF
Chlamydia trachomatis            QFAQVGLDTSWQVVFDPGIGF--GKTPVQSMLLMDGV
Chlamydia muridarum              TFAQIGLDTSWQVVFDPGIGF--GKTPVQSMQLMEGV
Chlamydophila caviae             AFVALGLRQD-QIIFDPGIGF--GTTQIQALNVLHKM
Chlamydia psittaci               AFVALGLSKD-QIIFDPGIGF--GTTPIQALNVLHRM
Chlamydia felis                  AFTALGLNLD-QIIFDPGIGF--GTSPIQALHVLHRM
Chlamydia avium                  FCDHLGLDCS-QIIFDPGIGF--GTTSIQAMYILQKM
Chlamydia gallinacea             FCDRLGLDCS-QIIFDPGIGF--GTTSIQAMRILREM
Chlamydophila pneumoniae         MFSDVGLNAN-QVIFDPGIGF--GKGAAQSLATLYEI
Parachlamydia acanthamoebae      ELKKNGVKEE-RIILDPGIGF--GKTVDDNLQIIQNL
Waddlia chondrophila             ALQKAGIKEN-NIIIDPGIGF--GKTVEDNYQILHNL
```

Supplementary Data 3 – Detail of the multiple sequence alignment region containing the two-amino acid signature motif including Sul sequences and FolP sequences from members of the *Rhodobiaceae*, the *Leptospiraceae* and the Chlamydiae.
