# Supplementary material for: Origin of the Mobile Di-Hydro-Pteroate Synthase Gene Determining Sulfonamide Resistance in Clinical Isolates
Source: Front Microbiol. 2019 Jan 10;9:3332. doi: 10.3389/fmicb.2018.03332 (PMC6335563; doi:10.3389/fmicb.2018.03332)
Supplement: Supplementary file 9 [file Data_Sheet_4.PDF]

Supplementary Material

Origin of the mobile di-hydro-pteroate synthase gene determining sulfonamide resistance in clinical isolates

Miquel Sánchez-Osuna[1], Pilar Cortés[1], Jordi Barbé[1*], Ivan Erill[2*]

*** Correspondence:** Corresponding Authors: jordi.barbe@uab.cat; erill@umbc.edu

Supplementary Data 4 – Unrooted Neighbor-Joining tree of Sul/FolP homologs. Branch support values are provided as the percent of bootstrap replicates in which the branching was observed. Support values are only shown for branches with at least 80% support. The cluster of Actinobacteria duplicated *folP* gene products that were removed from further analysis is indicated by the shaded ellipse.

frontiers

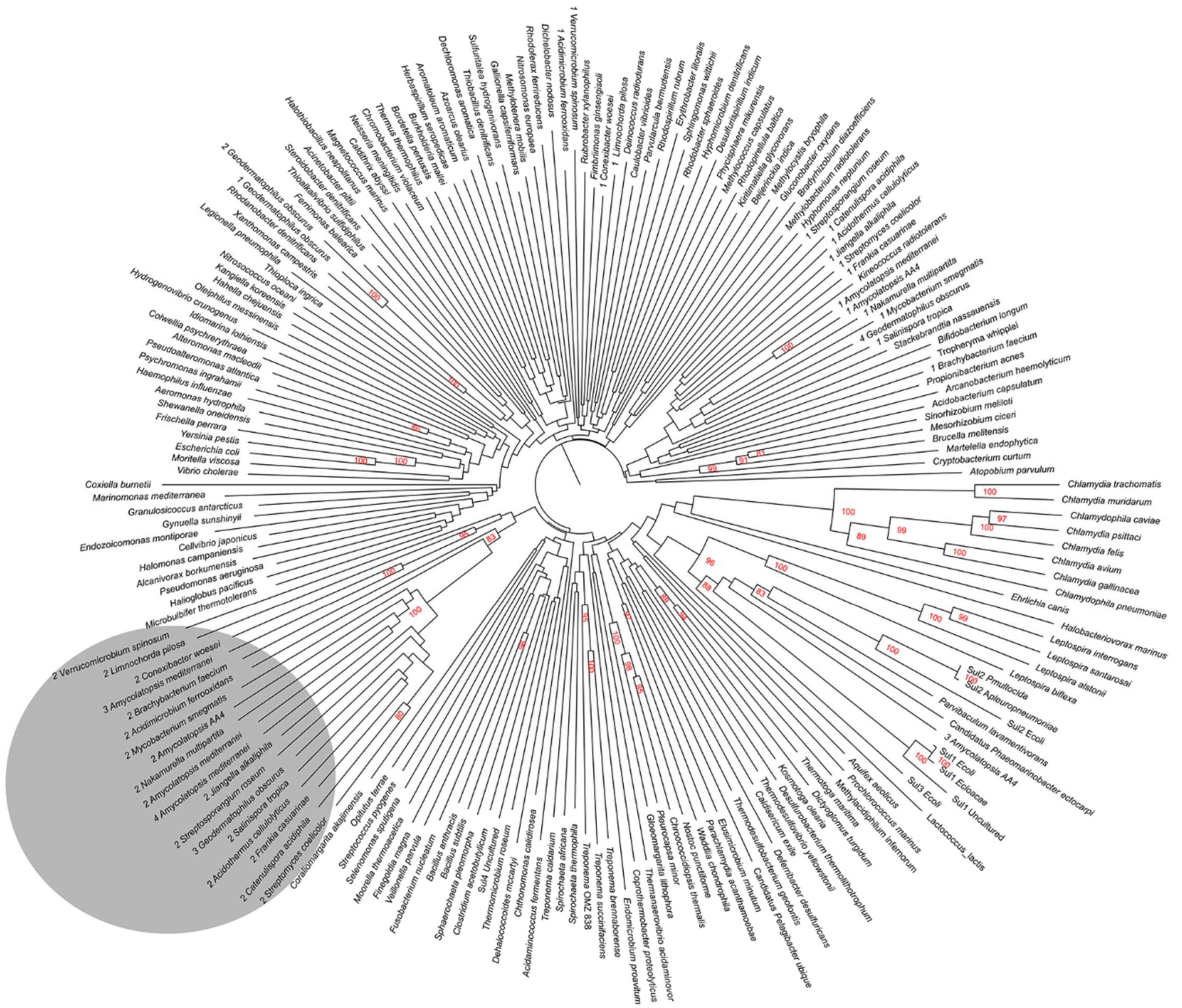

1 Verrucomicrobium spinosum
1 Acidimicrobium ferrooxidans
Dichelobacter nodosus
Rhodoferax ferrireducens
Nitrosomonas europaea
Methylotenera mobilis
Gallionella capsiferriformans
Sulfuritalea hydrogenivorans
Thiobacillus denitrificans
Dechloromonas aromatica
Azoarcus olearius
Aromatoleum aromaticum
Herbaspirillum seropedicae
Burkholderia mallei
Bordetella pertussis
Thermus thermophilus
Chromobacterium violaceum
Neisseria meningitidis
Caldimonas manganoxidans
Magnetococcus marinus
Halothiobacillus neapolitanus
Acinetobacter pittii
Steroidobacter denitrificans
Thioalkalivibrio sulfidiphilus
Ferrimonas balearica
2 Geodermatophilus obscurus
1 Geodermatophilus obscurus
Rhodanobacter denitrificans
Xanthomonas campestris
Legionella pneumophila
Thioploca ingrica
Nitrosococcus oceani
Kangiella koreensis
Hahella chejuensis
Oleiphilus messinensis
Hydrogenovibrio crunogenus
Idiomarina loihiensis
Colwellia psychrerythraea
Alteromonas macleodii
Pseudoalteromonas atlantica
Psychromonas ingrahamii
Haemophilus influenzae
Aeromonas hydrophila
Shewanella oneidensis
Frischella perrara
Yersinia pestis
Escherichia coli
Moritella viscosa
Vibrio cholerae
Coxiella burnetii
Marinomonas mediterranea
Granulosicoccus antarcticus
Gynuella sunshinyii
Endozoicomonas montiporae
Cellvibrio japonicus
Halomonas campaniensis
Alcanivorax borkumensis
Pseudomonas aeruginosa
Halioglobus pacificus
Microbulbifer thermotolerans
2 Verrucomicrobium spinosum
2 Limnochorda pilosa
2 Conexibacter woesei
3 Amycolatopsis mediterranei
2 Brachybacterium faecium
2 Acidimicrobium ferrooxidans
2 Mycobacterium smegmatis
2 Amycolatopsis AA4
2 Nakamurella multipartita
2 Amycolatopsis mediterranei
4 Amycolatopsis mediterranei
2 Jiangella alkaliphila
2 Streptosporangium roseum
3 Geodermatophilus obscurus
2 Salinispora tropica
2 Acidothermus cellulolyticus
2 Frankia casuarinae
2 Catenulispora acidiphila
2 Streptomyces coelicolor
Coraliomargarita akajimensis
Opitutus terrae
Streptococcus pyogenes
Selenomonas sputigena
Moorella thermoacetica
Finegoldia magna
Veillonella parvula
Fusobacterium nucleatum
Bacillus anthracis
Bacillus subtilis
Sphaerochaeta pleomorpha
Clostridium acetobutylicum
Sul4 Uncultured
Thermomicrobium roseum
Dehalococcoides mccartyi
Chthonomonas calidirosea
Acidaminococcus fermentans
Treponema caldarium
Spirochaeta africana
Spirochaeta thermophila
Treponema OMZ 838
Treponema succinifaciens
Treponema brennaborense
Endomicrobium proavitum
Coprothermobacter proteolyticus
Thermanaerovibrio acidaminovorans
Gloeomargarita lithophora
Pleurocapsa minor
Chroococcidiopsis thermalis
Nostoc punctiforme
Waddlia chondrophila
Parachlamydia acanthamoebae
Elusimicrobium minutum
Thermodesulfobacterium geofontis
Candidatus Pelagibacter ubique
Caldisericum exile
Deferribacter desulfuricans
Thermodesulfovibrio yellowstonii
Desulfurobacterium thermolithotrophum
Kosmotoga olearia
Thermotoga maritima
Dictyoglomus turgidum
Methylacidiphilum infernorum
Prochlorococcus marinus
Aquifex aeolicus
Lactococcus_lactis
Sul3 Ecoli
Sul1 Uncultured
Sul1 Ecloacae
Sul1 Ecoli
3 Amycolatopsis AA4
Candidatus Phaeomarinobacter ectocarpi
Parvibaculum lavamentivorans
Sul2 Ecoli
Sul2 Apleuropneumoniae
Sul2 Pmultocida
Leptospira biflexa
Leptospira alstonii
Leptospira santarosai
Leptospira interrogans
Halobacteriovorax marinus
Ehrlichia canis
Chlamydophila pneumoniae
Chlamydia gallinacea
Chlamydia avium
Chlamydia felis
Chlamydia psittaci
Chlamydophila caviae
Chlamydia muridarum
Chlamydia trachomatis
Atopobium parvulum
Cryptobacterium curtum
Martelella endophytica
Brucella melitensis
Mesorhizobium ciceri
Sinorhizobium meliloti
Acidobacterium capsulatum
Arcanobacterium haemolyticum
Propionibacterium acnes
1 Brachybacterium faecium
Tropheryma whipplei
Bifidobacterium longum
Stackebrandtia nassauensis
1 Salinispora tropica
4 Geodermatophilus obscurus
1 Mycobacterium smegmatis
1 Nakamurella multipartita
1 Amycolatopsis AA4
1 Amycolatopsis mediterranei
Kineococcus radiotolerans
1 Frankia casuarinae
1 Streptomyces coelicolor
1 Jiangella alkaliphila
1 Acidothermus cellulolyticus
1 Catenulispora acidiphila
1 Streptosporangium roseum
Hyphomonas neptunium
Methylobacterium radiotolerans
Bradyrhizobium diazoefficiens
Gluconobacter oxydans
Methylocystis bryophila
Beijerinckia indica
Rhodopirellula baltica
Kiritimatiella glycovorans
Methylococcus capsulatus
Phycisphaera mikurensis
Desulfurispirillum indicum
Hyphomicrobium denitrificans
Rhodobacter sphaeroides
Sphingomonas wittichii
Erythrobacter litoralis
Rhodospirillum rubrum
Parvularcula bermudensis
Caulobacter vibrioides
Deinococcus radiodurans
1 Limnochorda pilosa
1 Conexibacter woesei
Fimbriimonas ginsengisoli
Rubrobacter xylanophilus
