# Supplementary material for: Origin of the Mobile Di-Hydro-Pteroate Synthase Gene Determining Sulfonamide Resistance in Clinical Isolates
Source: Front Microbiol. 2019 Jan 10;9:3332. doi: 10.3389/fmicb.2018.03332 (PMC6335563; doi:10.3389/fmicb.2018.03332)
Supplement: Supplementary file 12 [file Data_Sheet_7.PDF]

Supplementary Material

Origin of the mobile di-hydro-pteroate synthase gene determining sulfonamide resistance in clinical isolates

Miquel Sánchez-Osuna[1], Pilar Cortés[1], Jordi Barbé[1*], Ivan Erill[2*]

*** Correspondence:** Corresponding Authors: jordi.barbe@uab.cat; erill@umbc.edu

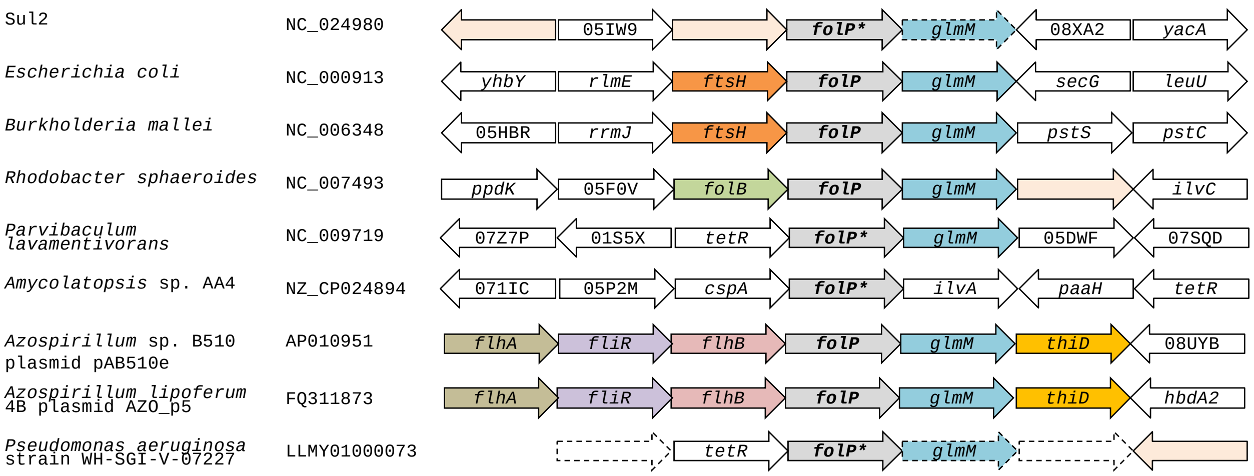

Supplementary Data 7 – Schematic representation of the genetic environment of *sul2* genes, similar arrangements in chromosomally-encoded *folP* genes of the Gammaproteobacteria, Betaproteobacteria and Alphaproteobacteria, and arrangements in other putative mobilization instances of the *folP* gene. Arrow boxes indicate coding regions. When available, gene names or NOG identifiers are provided. Boxes for *folP* genes containing the two-amino acid insertion are designated as *folP**.
